# The Effect of Alcohol Intoxication on Mortality of Blunt Head Injury

**DOI:** 10.1155/2014/619231

**Published:** 2014-08-04

**Authors:** Hsing-Lin Lin, Tsung-Ying Lin, Kwan-Ming Soo, Chao-Wen Chen, Liang-Chi Kuo, Yen-Ko Lin, Wei-Che Lee, Chih-Lung Lin

**Affiliations:** ^1^Division of Trauma, Department of Surgery, Kaohsiung Medical University Hospital, Kaohsiung Medical University, Kaohsiung 807, Taiwan; ^2^Department of Emergency Medicine, Kaohsiung Medical University Hospital, Kaohsiung Medical University, Kaohsiung 807, Taiwan; ^3^Faculty of Medicine, College of Medicine, Kaohsiung Medical University, Kaohsiung 807, Taiwan; ^4^Division of Neurosurgery, Department of Surgery, Kaohsiung Medical University Hospital, 100 Tzyou 1st Road, Kaohsiung 807, Taiwan

## Abstract

Alcohol is found to have neuroprotection in recent studies in head injuries. We investigated the association of blood alcohol concentration (BAC) with mortality of patients with blunt head injury after traffic accident. All patients sustaining blunt head injury caused by traffic accident brought to our emergency department who had obtained a brain computed tomography scans and BAC were analyzed. Patients with unknown mechanisms, transfers from outside hospitals, and incomplete data were excluded. Logistic regression was used to identify independent predictors of mortality. During the study period, 3,628 patients with brain computed tomography (CT) were included. Of these, BAC was measured in 556 patients. Patients with the lowest BAC (less than 8 mg/dl) had lower mortality; intoxicated patients with BAC between 8 and less than 100 mg/dl were associated with significantly higher mortality than those patients in other intoxicated groups. Adjusted logistic regression demonstrated higher BAC group and Glasgow coma scale (GCS) scores, and lower ISS and age were identified as independent predictors of reduced mortality. In our study, we found that patients who had moderate alcohol intoxication had higher risk of mortality. However, higher GCS scores, lower ISS, and younger age were identified as independent predictors of reduced mortality in the study patients.

## 1. Introduction

Alcohol consumption is considered as a risk factor of mortality in patient with traumatic brain injury (TBI). Management of TBI is to reduce the secondary brain injury and protect the remaining living brain tissue from ischemia. Many medicines such as sedatives, mannitol (C_6_H_8_(OH)_6_), and hypertonic saline are used in the treatment of increased intracranial pressure caused by TBI with diuretic effect. Alcohol also has the similar influence to increase urine output. Therefore, it might provide some protection after brain injury.

In previous studies, many risk factors associated with mortality of TBI have been identified. The effect of blood alcohol concentration (BAC) on the outcomes of TBI still remains controversial [1–6]. Ethanol (C_2_H_5_OH) is the type of alcohol found in alcoholic beverages. Its diuretic effect may decrease the blood pressure of the traumatic patients during resuscitation but may also reduce intracranial pressure. In studies using animal models, there is still no consensus on the benefit of alcohol in neuroprotection, with some negative and positive results or even no effect [7–11]. Similarly, clinical studies have shown conflicting results of the BAC effect and most of the studies have focused on moderate to severe brain injury [1–6].

The effect of alcohol use on trauma outcome has been studied with mixed results. Nevertheless, few studies have focused on the outcome in alcohol-intoxicated patients with TBI [5]. The objective of this study was to investigate the mortality of blunt head injury in patients with acute intoxication by alcohol.

## 2. Patients and Methods

### 2.1. Patients and Setting

In this retrospective study, the trauma registry at Kaohsiung Medicine University Hospital, an urban level I trauma center, was used to identify all trauma patients evaluated from January 2007 to May 2011. The hospital provides medical center care with 1200 beds and has an average of 8,000 ED visits per month, including 1,200 trauma patients. All patients sustaining blunt head injury after traffic accident who had brain-computed tomography (CT) were selected for this study. Patients without brain CT were excluded because their head injury was too minor to be of concern or too severe to survey. Patients transferred from other hospitals were also excluded. The study protocol was approved by the Institutional Review Board of Kaohsiung Medical University.

Patient demographic data and variables including age, gender, mechanism of injury, injury severity score (ISS), laboratory value (BAC), admission vitals blood pressures, and Glasgow coma scale (GCS) were collected from the trauma data bank. Mortality was defined as in-hospital death. The ISS was calculated by trauma registry staff after discharge or death in each patient. BAC tests at our facility are measured at the discretion of the attending physician at the ED when a patient with head injury is suspected of being intoxicated. Obtained brain CT in the blunt head injury patient followed the protocols of the Bureau of National Health Insurance, Department of Health, and was strictly monitored. In this study, we considered BAC of more than 8 mg/dL as positive to be intoxication because BAC of more than 8 mg/dL would have skills impairment based on a review article by the U.S. Department of Transportation and National Highway Traffic Safety Administration after reviewing 112 articles from 1981 to 1997. All patients' mortality was caused by trauma-related death.

### 2.2. Statistical Analysis

The BAC of study population was then stratified into four levels: none (less than 8 mg/dL), low (8 to less than 100 mg/dL), moderate (100 to less than 230 mg/dL), and high (≥230 mg/dL) [1]. A group of patients not tested for BAC were categorized as a compared group, and the patients with BAC test with level less than 8 mg/dL were added as the none group. Then, the mortality of these patients was compared for differences in baseline clinical and demographic characteristics using bivariate analysis. Categorical variables were compared by the *χ* ^2^ or Fisher exact test, and continuous variables were compared using Student's* t*-test or Mann-Whitney rank-sum test. One-way analysis of variance was used to test the means of two or more groups with post hoc tests between groups. To investigate the association between BAC and mortality and to estimate the adjusted odds ratio (OR) for death, multivariable logistic regression analysis was performed to adjust for statistically and clinically relevant confounding factors. All factors that on analysis were significant with a *P* value < 0.2 were entered into a logistic regression to determine independent predictors of mortality. A value of *P* < 0.05 was considered statistically significant. Data analyses were performed using SPSS software (version 15; SPSS Inc., Chicago).

## 3. Results

During the study period, a total of 8,724 patients with blunt head injury who obtained brain CT were admitted to the ED at our hospital. After excluding patients with unknown mechanisms, transfers from outside hospitals, and those with incomplete data, 3,628 patients were included in the study. Of these, BAC was measured in 556 (15.3%) patients. BAC levels were positive in 60.0% (334) and negative (less than 8 mg/dL) in 39.9% (222) of the blunt head injury patients. In Table 1, a comparison of patient characteristics and mortality between patients with blunt head injury tested and not tested for BAC is demonstrated. Patients who were tested for BAC were more often male (75.5% versus 52.2%, *P* < 0.001), had lower age (41.09 ± 15.97 versus 50.05 ± 23.36, *P* < 0.001), more often had lower GCS (11.47 ± 4.31 versus 14.24 ± 2.34, *P* < 0.001) and systolic blood pressure (SBP) (137.8 ± 28.4 versus 145.0 ± 28.5 mmHg, *P* < 0.001), and had higher ISS (13.6 ± 9.4 versus 8.3 ± 7.3, *P* < 0.001) and mortality (6.8% versus 3.4%, *P* < 0.001) when compared with the patients who were not tested for BAC.

All variables in the univariate analysis with *P* < 0.2 were analyzed in a logistic regression enter model (Table 2). BAC, ISS, GCS, age, gender, and SBP were all entered into the logistic regression model as confounding factors with “enter” model. BAC groups (*P* = 0.007, OR = 0.567, 95% CI 0.376–0.855) and GCS (*P* = 0.001, OR = 0.727, 95% CI 0.683–0.774), ISS (*P* = 0.001, OR = 1.130, 95% CI 1.099–1.163), and age (*P* < 0.001, OR = 1.048, 95% CI 1.033–1.065) were identified as independent predictors of reduced mortality of the study patients. The model adequately fitted the data with the Hosmer-Lemeshow statistics, indicating a good fit (*P* = 0.281), while 47.6% of the variation was explained by the model (Nagelkerke *R* Square).

In Table 3, the percentages of different BAC levels in mortality and ISS of patients are shown. Mortality was higher in the group of BAC between 8 and less than 100 mg/dL than other intoxicated groups (*P* < 0.001). Although ISS was higher in the groups of BAC-positive than the none group (*P* < 0.001), there were no significant differences between the groups with BAC higher than 8 mg/dL with post hoc analysis.

## 4. Discussion

In this study, we found that, in patients with blunt head injury, patients in the none group had less severity and mortality, and the mortality was higher in patients with BAC between 8 and less than 100 mg/dL group than other intoxicated groups.

In our study, those in the non-BAC-tested group had lower ISS and lower mortality. The finding is similar to the study by Salim et al. The ISS was also lower in their study patients who were not BAC-tested when compared with the BAC-tested (ISS: not tested versus tested, 20.5 ± 9.0 versus 22.7 ± 10.2, *P* < 0.001). However, they reported that mortality was higher in the patients without BAC test (mortality: tested versus not tested, 8.9 versus 9.6, *P* < 0.001) [12]. In our study, we found that BAC-tested patients had higher mortality than non-BAC-tested patients. If patients were drunk, their consciousness might be affected. Those who had BAC-test were more drowsy or unclear of consciousness. The ability to protect themselves might be impaired. Thus, when an accident happened, they might have more severe injuries than those without alcohol consumption. We think that the difference of mortality was due to the patient's selection. We included all head injury patients; most were mild to moderate head injury (GCS 13.8 ± 2.9), but their patients were moderate to severe traumatic brain injury (head abbreviated injury score more than 3).

According to the study by Hadjibashi et al. [13], higher BAC had lower rates of pneumonia after TBI. In the study by Tien et al. [14], ISS and mortality were higher in the non-BAC-tested group than in the group with BAC less than 230 mg/dL in moderate and severe TBI patients. However, when compared with the group of BAC more than 230 mg/dL, the none group had lower mortality. In our study, we did not findhigher mortality in the higher BAC patients, because our patients with higher BAC had relatively low ISS than other studies. However, our results were similar to the study reported by Porter, who found a trend to decreased injury severity with the presence of alcohol [15]; thus, higher BAC-intoxicated patients might result in lower speed accident, which might be a reason of lower ISS in our patients due to the punishment of the law of drinking driver in Taiwan is very severe. People are prone to drive slowly to avoid being caught by law enforcers. In addition, most of our patients had motorcycle-related injury, which might result in the lower ISS compared to other studies.

It is a surprising finding that our patients with BAC more than 100 mg/dL had lower mortality than BAC between 8 and less than 100 mg/dL group. At present, the mechanisms whereby alcohol intoxication reduces the mortality of head injury are still unknown. The diuretic effect might reduce the increase of intracranial pressure after trauma. In addition, brain atrophy in some chronic alcoholism cases may have more room for hematoma to prevent increased intracranial pressure, which might be a potential cause. Besides, in some animal studies, low to moderate doses of BAC have neuroprotective effect and even improve neurologic outcome [7, 11, 16, 17]. Further study on the relationship between the level of BAC and mortality is needed to elucidate the mechanism.

Although this is a single-institution study and may reflect the characteristics only of local patients, our hospital is located in the central area of the city, and most patients were brought to the hospital directly without diversion. The average time of patients being brought by Emergency Medical Technicians (from scene tohospital) was short (less than one hour, data no shown); therefore, our patients may represent the population of head injury from mild to moderate in the urban area, which is the strength of this study. Nevertheless, as a retrospective study, there are several limitations where the results may be exaggerated by confounders and biases. The BAC test is not necessary in every trauma patient; nevertheless, we included all patients with blunt head injury with brain CT obtained in the ED, which resulted in only 15.3% of the blunt head injury patients being found to have the BAC test. However, alcohol is not a common drink at the table (in Chinese culture) and the government enforces the law of drinking and driving in our country strictly, so there are few trauma patients brought to hospital with intoxication. Although whether the patient was an acute or chronic alcohol abuser or had dependence cannot be determined, we analyzed the BAC with actual level, which is not available in some large nationwide data banks. Besides, we analyzed all patients with head injury who obtained brain CT at the ED; this created no difficulty to apply our results to all patients with blunt head injury. Secondly, the demographic data in those patients who had BAC drawn were significantly different from those patients without BAC drawn (Table 1). However, a higher mortality and ISS were seen in those patients with BAC between 8 and less than 100 mg/dL as compared with BAC more than 100 mg/dL, which was a surprising result. In this study, the detailed neurological and functional outcomes were not obtained, with only mortality being analyzed. Even so, we evaluated GCS as the neurological deficit and would be the strength of this study. The alcohol in the patient's blood may be eliminated via metabolism. In our study, the average time of a patient to arrive at our institution is within one hour, which minimized the bias of metabolism. Despite these limitations, our study provides further information of the neuroprotection of alcohol in the head injury to the growing literature.

## 5. Conclusion

The study found that patients with blunt head injury had higher mortality in mild alcohol intoxication (8 to less than 100 mg/dL) than other intoxicated groups. However, other relevant factors, including ISS, GCS, and age also have considerable impact on mortality. The mechanisms by which moderate to severe alcohol intoxication reduces the mortality rate when compared to mild intoxicated patients with blunt head injury still need further study for confirmation.

## Figures and Tables

**Table 1:** Comparison of demographics and mortality of patients according to BAC availability.

	All patients (*n* = 3628)	Tested for BAC (*n* = 556)	Not tested for BAC (*n* = 3072)	*P*
Age (yr), mean ± SD	48.68 ± 22.62	41.09 ± 15.97	50.05 ± 23.36	<0.001
Gender (Male%)	2025 (55.8%)	420 (75.5%)	1603 (52.2%)	<0.001
GCS	13.8 ± 2.9	11.5 ± 4.3	14.2 ± 2.3	<0.001
SBP	144.0 ± 28.6	137.8 ± 28.4	145.0 ± 28.5	<0.001
ISS, mean ± SD	9.1 ± 7.9	13.6 ± 9.4	8.3 ± 7.3	<0.001
Mortality	3.9% (141/3628)	6.8% (38/556)	3.4% (103/3072)	<0.001

ISS: Injury Severity Score; GCS: Glasgow coma scale; SBP: systolic blood pressure. *P* value resulted from comparing tested and not tested for BAC.

**Table 2:** Independent risk factors associated with mortality of the study patients (*n* = 3628).

	OR	95% CI	*P*
BAC	0.567	0.376–0.855	0.007
ISS	1.130	1.099–1.163	0.001
GCS	0.727	0.683–0.774	0.001
Age	1.048	1.033–1.065	<0.001
Gender	0.779	0.461–1.315	0.350
SBP	1.004	0.996–1.012	0.326

The BAC of study population was stratified into four levels: none (less than 8 mg/dL), low (8 to less than 100 mg/dL), moderate (100 to less than 230 mg/dL), and high (≥230 mg/dL). Those whose BAC was not tested were stratified as none.

**Table 3:** The comparisons of BAC levels by mortality and ISS (*n* = 3628).

	Mortality (*n* = 141/3628) (*P* ^#^ < 0.001)	ISS (P* < 0.001)
BAC (mg/dL)		
Less than 8	3.7% (123/3296)	8.77 ± 7.66
8 to less than 100	9.8% (4/41)	14.10 ± 8.52
100 to less than 230	4.5% (9/165)	13.05 ± 9.77
≥230	5.4% (5/126)	11.37 ± 8.47

*P* ^#^: Chi-square. There is a statistical significant difference of mortality among BAC levels. P*: one-way analysis of variance. There is a statistical significant difference of ISS among BAC levels.
